# Human Herpesvirus Sequencing in the Genomic Era: The Growing Ranks of the Herpetic Legion

**DOI:** 10.3390/pathogens8040186

**Published:** 2019-10-12

**Authors:** Charlotte J. Houldcroft

**Affiliations:** 1 Department of Medicine, Addenbrooke’s Hospital, University of Cambridge, Cambs CB2 0QQ UK; ch504@cam.ac.uk; 2 Parasites and Microbes, Wellcome Sanger Institute, Wellcome Genome Campus, Hinxton, Cambs CB10 1SA, UK

**Keywords:** genomics, herpesviruses, DNA viruses, population genetics, clinical sequencing

## Abstract

The nine human herpesviruses are some of the most ubiquitous pathogens worldwide, causing life-long latent infection in a variety of different tissues. Human herpesviruses range from mild childhood infections to known tumour viruses and ‘trolls of transplantation’. Epstein-Barr virus was the first human herpesvirus to have its whole genome sequenced; GenBank now includes thousands of herpesvirus genomes. This review will cover some of the recent advances in our understanding of herpesvirus diversity and disease that have come about as a result of new sequencing technologies, such as target enrichment and long-read sequencing. It will also look at the problem of resolving mixed-genotype infections, whether with short or long-read sequencing methods; and conclude with some thoughts on the future of the field as herpesvirus population genomics becomes a reality.

## 1. Introduction

**Figure 1:**
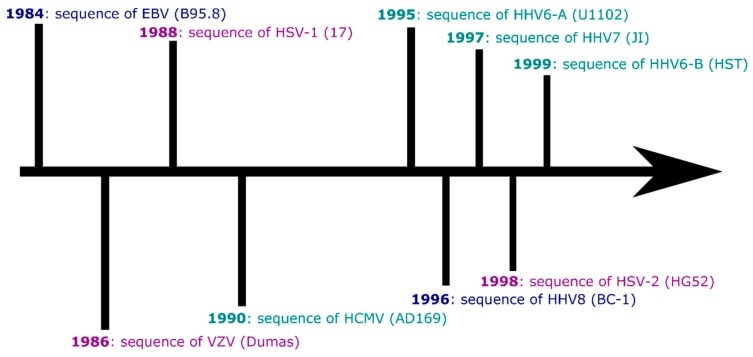
A timeline of human herpesvirus genome sequence publication (strain name in brackets). Alphaherpesviruses are marked in magenta; betaherpesviruses are marked in teal; gammaherpesviruses are marked in dark blue.

There are nine currently recognised human herpesviruses. All share the classic herpesvirus life cycle of causing primary infection (typically in childhood) before establishing latency within particular cell subsets, awaiting periods of reduced host immunity to reactivate and go on to infect new hosts. Humans carry herpesviruses in three subfamilies: the alphaherpesvirinae, betaherpesvirinae and gammaherpesvirinae. Within and between these subfamilies, the human herpesviruses have tropisms for a range of tissues (lymphocytes, epithelia) and have genomes of varying sizes (from approximately 125,000 to 235,000 base pairs in length). Some of the human herpesviruses have strong associations with specific diseases (lymphomas, symptomatic primary infection), while others are less clearly linked to disease in adulthood. All of the human herpesviruses were sequenced at the whole-genome level over a period of around 15 years (Figure 1), and following this ‘Age of Discovery’, some have experienced a trickle of new genomic data (HHV7) and other viruses a flood (Epstein-Barr virus).

This review is not intended to be an exhaustive history of the sequencing of herpesviruses or a detailed picture of the entire field of herpesvirus genomics. Instead, I hope to provide vignettes of where genomics for each virus currently stands, and interesting future directions herpesvirus genomics may take.

## 2. Sequencing Herpesviruses: From PCR to High-Throughput and Target Enriched Sequencing

The human herpesviruses range in length from ~125kbp (varicella-zoster virus (VZV)) to ~235kbp (human cytomegalovirus (HCMV)). This makes their genomes much less amenable to over-lapping PCR or rolling-circle amplification [1] for sequencing than other double-stranded DNA viruses such as adeno [2], polyoma [3] or papillomaviruses [4], which are all considerably smaller (although over-lapping PCR amplicon sequencing approaches have been used for eg HCMV [5]).

After the sequencing and publication of the reference sequences of the nine human herpesviruses, there was gradual accrual of further genome sequences for the next ~25 years. In 2011, there were in total 29 whole human herpesvirus genomes in GenBank [6]. The B95.8 EBV sequence was published in 1984 (Figure 1), and until 2013, there were ten published whole EBV genome sequences. A similar situation persisted for VZV until 2009, driven in part by interest in sequencing vaccine strains of the virus [7]. 2010 can be seen as an important year for cytomegalovirus genomics, transitioning to sequencing and comparison of multiple HCMV strains, including from clinical material [8]. What has changed since 2011 (Table 1) to increase the number of herpesvirus genomes from tens to thousands?

I argue that the changes to sequencing herpesvirus genomes that have transformed this area of science can be related to three factors over the last decade: the change from Sanger (dideoxy) to high-throughput sequencing (HTS), most notably Illumina short-read technology; the use of target-enrichment technologies [6]; and the falling cost of sequencing [9].

These changes have been especially important for sequencing directly from clinical samples. In cultured virus isolates, it is possible to generate high concentrations and relatively large volumes of viral genome material for sequencing, which can be further amplified in over-lapping PCR amplicons (with potentially hundreds required to tile a herpesvirus genome) or which can provide material to be sequenced directly. Both approaches increase the signal (viral genome) to noise (host genome) ratio of a herpesvirus sequencing project. However, for some herpesviruses there are well-known problems of rapid genomic adaptation to culture, most notably in HCMV [10]. This is also seen in human herpesvirus 6A (HHV6A) [11]. Sequencing without PCR amplification more accurately represents the diversity present in the original sample [12]. However sequencing directly from clinical material is only possible when virus loads are high [13], and where there is abundant clinical material and high read depths are achieved, because the data is likely to be dominated by host reads [6]. A greater depth of sequence (with associated reagent and analysis costs/time) is thus required to generate a viral consensus genome at a given depth [14].

Target enrichment technology such as IDT, MyBaits or SureSelect DNA and RNA oligonucleotide baits have driven our ability to sequence from clinical samples, avoiding the problem of culture artefacts in sequence data [14]. By binding to the target viral DNA and allowing host DNA to be largely washed away, target enrichment can increase the proportion of reads from the virus of interest, reduce the size of each sample library, and in turn allow greater multiplexing of samples in each sequencing reaction and reduced costs [15]. Batching of samples typically means this approach is employed by very large clinical centres or by laboratories with a specialist focus on herpesvirus sequencing where extensive sample collections can be built up [16,17,18].

The final change which has favoured herpesvirologists (and genomics in general) is the falling cost of sequencing itself [9]. The lower per-base price of sequencing is making viral population genomic studies a reality, as genomes from across the world can be compared [19,20,21,22]. However, as with target enrichment, the requirement to multiplex virus genome sequencing at high depth to maximise the cost reductions increasingly concentrates sequencing into larger laboratories [23]. Long-read sequencing technologies will be a further disruptive influence (discussed below).

### 2.1. Alphaherpesviruses

Humans carry two simplex viruses, herpes simplex virus 1 (HSV-1) and herpes simplex virus 2 (HSV-2), unlike our closest living relatives chimpanzees, bonobos and gorillas which have a single, oral simplex virus species each [24]. Both human viruses are able to cause oral and genital infection, as well as encephalitis [25] and potentially severe neonatal disease [26]. HSV-1 is more widespread globally [27], and HSV-2 reaches its highest seroprevalence in sub-Saharan Africa [28]. Sequencing has unsurprisingly focused on severe symptomatic HSV infections such as genital herpes and neonatal disease, in part because these cases provide higher virus loads and readily available clinical material for sequencing, as well as the greatest burden of disease compared to more prevalent but mild presentations such as cold sores.

#### 2.1.1. Herpes Simplex Virus 1

Herpes simplex virus 1 is the ancestral human HSV [24,29]. Originally associated with infections of the oral mucosa [27], HSV-1 is now an important cause of genital herpes worldwide. In USA, HSV-1 has overtaken HSV-2 as the leading cause of new cases of genital herpes [30].

The HSV-1 strain which provided the reference sequence was isolated in 1972, and its genome was sequenced via Sanger sequencing of plasmids, later supplemented by Illumina sequencing of viral DNA [31]. Since then, sequencing of HSV-1 genomes has sought to understand global diversity in HSV-1 [22,32], supporting an Out-of-Africa hypothesis for the spread of the virus [33,34]. Diversity has also been studied at a finer grain in specific populations, ie the Finnish [35].

High-throughput sequencing has allowed researchers to elucidate the histories of commonly used HSV-1 strains KOS63 and KOS79, which were isolated independently from the same individual who had experienced a dual-strain infection [36]. HSV-1 genomics has also been applied to the transmission of HSV-1 within families [37] and the evolution of the virus in individuals over time [38].

HSV-1 sequencing studies have also shed light on the debate over direct metagenomic versus culture enriched versus target enriched sequencing discussed earlier in this paper. Low-passage cultured HSV-1 isolates were compared to paired virus from the same patient sequenced directly from clinical material. Cultured virus isolates underwent minimal change, which has allayed worries that HSV-1 undergoes significant and rapid changes in cell culture at low passage numbers [15].

#### 2.1.2. Herpes Simplex Virus 2

Appropriately for the virus which is classically thought of as the cause of genital herpes, even if that epidemiological pattern is changing, the reference sequence for HSV-2 (strain HG52) was isolated from a woman with genital herpes. The virus was sequenced from cloned plasmids, with later Illumina sequencing (as with the HSV-1 RefSeq) [39].

HSV-2 was originally a chimpanzee oral pathogen than jumped the species barrier around 1.6 million years ago [24], perhaps via an intermediate hominin host [29], and it is currently unclear when and how HSV-2 specialised in the human genital niche. Deliberate attempts to characterise diversity from around the world [40] have been powerful tools for analysing the evolutionary history of HSV-2 [33,41,42], revealing ancient recombination events in the majority of HSV-2 genomes. These recombinant strains, which represent the majority of HSV-2 found outside Africa, are thought to be better adapted to infecting human hosts, hence their wide geographic spread compared to non-recombinant strains. The genesis of new recombinant HSV-2 strains has even been detected in real time in genitally co-infected patients [16]. As with HSV-1, there has also been an important focus on sequencing HSV-2 strains associated with neonatal disease [26].

#### 2.1.3. Varicella-Zoster Virus

Varicella-zoster virus is typically a childhood infection, spread by exposure to virus particles through coughing, skin to skin contact or aerosolized virus particles from the skin rashes caused by primary infection (varicella, chickenpox) or reactivation (zoster, shingles) [43]. There has been a drive to understand the population genetics of circulating VZV in order to better understand the morbidity caused by zoster because of its association with post-herpetic neuralgia [44] and stroke [45].

VZV has relatively low genomic diversity compared to other herpesviruses [46,47], which makes bait-based sequencing of VZV easier and has in part fuelled the success of sequencing studies. Insights from recent sequencing studies have been reviewed in-depth [48] but highlights include studies which have targeted the evolution of the live-attenuated vaccine stain vOka both in the lab [7] and in clinical use [49]. Whole genome sequencing of VZV has been important in refining SNP-based genotyping systems which help to distinguish between natural and vaccine derived infections and reactivations [50]. Population-level sequencing and studies of the mutation rate of VZV between primary infection and reactivation have also addressed the controversy of whether current patterns of circulating VZV diversity reflect an Out-of-Africa co-divergence of populations and virus lineages; or whether circulating VZV has an origin in Neolithic Europe [21,49]. In the latter example, only ancient VZV DNA is likely to settle this question, but it demonstrates the ability of pathogen sequencing studies to challenge our assumptions about the history of human diseases.

### 2.2. Betaherpesviruses

#### 2.2.1. Cytomegalovirus

Human cytomegalovirus has a rich sequencing history. Following the plasmid-library sequencing of AD169, sequencing methods have moved through bacterial artificial chromosome (BAC) and fragment cloning [51,52], sequencing of virion DNA [53] and multiple overlapping PCR amplicons [5], to low-passage culture [54] and target enrichment approaches [17,19,55].

The state of the HCMV sequencing landscape was recently and extensively reviewed elsewhere [56]. The field is certainly healthy, perhaps because of the association of HCMV with (sometimes severe) disease in transplant recipients and neonates [55,57], on-going difficulties in developing a vaccine [58] and the problem of drug resistance [59]. Drug resistance is also relevant to HSV1 [15] and VZV [60], but it is unclear if or when herpesvirus genome sequencing to detect antiviral resistance will become a clinically licensed diagnostic test [14].

The virus sequences generated to answer clinical questions can also be used to tackle questions such as the relationship between mixed-genotype infection, its role in drug resistance [17,61] and its association with HIV [57]. Clinical HCMV genomes have shed light on recent and ancient recombination events [17,19]; and currently circulating ‘natural knockouts’ with pseudogenised loci, which are still able to infect healthy individuals [17,54], show us the ability of HCMV to adapt to and exploit the health status of its human host.

#### 2.2.2. Human Herpesvirus 6A and 6B

Despite their similar names, human herpesviruses 6A (HHV6A) and 6B (HHV6B) are sufficiently different at the level of sequence similarity, immunological reactivity and perhaps disease association to be recognised as distinct species [62]. Both 6A and 6B integrate in to the sub-telomeric regions of human chromosomes, and following chromosomal integration, HHV6A and 6B strains can be inherited vertically if this integration occurs in a gamete [63]. These cases are described as inherited chromosomally integrated HHV6A/B (iciHHV6A/B). There have been more reported cases of iciHHV6B than iciHHV6A [64]. Carriage of iciHHV6 (A/B not specified) is associated with angina pectoris [65] and other kinds of heart failure [66].

Comparisons between these viruses, including both iciHHV6A/B and naturally circulating strains, have suggested that 6A is three-fold more diverse than 6B [67]. It remains to be seen whether increased sequencing will reveal strong geographic structure in the genome sequences of HHV6A or 6B that is comparable to eg VZV [21,68].

##### HHV6A

HHV6A genomics is slowly catching up to fellow betaherpesvirus HCMV (Table 1). The initial reference sequence U1102 was originally isolated in Uganda [69], and was followed by strain GS from the USA [70] and then strain AJ from the Gambia [71]. Further genomes have been recovered from clinical samples of primary infections and also iciHHV6A cases (see discussion of HHV6B below) [72]. It is unclear whether the diversity of both circulating and iciHHV6A genomes has been influenced by founder events in the human population. More strains and more pedigrees will need to be sequenced to identify if, and how long ago, these events occurred [63]. Work has also taken place to sequence laboratory reference material and low-passage strains, in order to examine variability in features such as the repetitive regions of the HHV6A and B genomes [73]. Associations between HHV6A and multiple sclerosis are likely to drive further interest in HHV6A genomic variation and its potential role in disease [74].

##### HHV6B

Studies focusing on the disease associations and evolution of iciHHV6A and B genomes have given us fascinating insights into *Roseolovirus* diversity and history. HHV6B is most closely associated with the childhood disease roseola infantum (exanthema subitem or ‘sixth disease’) [75]. Greninger and colleagues sequenced 125 genomes from sporadic primary infections and familial iciHHV6 pedigrees from Japan, USA and Uganda [76]. They identified that many individuals from the USA carry the same copy of iciHHV6B through a (human) founder event. Founder events leading to the spread of specific iciHHV6 haplotypes in Great Britain and elsewhere in Europe have also been identified. The last common ancestor of one of the European haplotypes was around 25,000 years ago. This molecular dating work allowed HHV6B to be firmly identified as a Pleistocene infectious disease (and almost certainly older) [63]. Further analysis revealed iciHHV6B in HapMap and 1000 Genomes samples and cell lines. Comparing these sequences to Z29 (the HHV6B reference sequence) showed that Z29 is an unusual and possibly unrepresentative strain of HHV6B, which may not be the ideal choice as the reference genome [67].

Sequencing of the HHV6B transcriptome has revealed differences in the RNAs expressed in latent HHV6B infection, iciHHV6B carriage, and plasma viraemia following haematopoietic stem cell transplantation [77]. Expression levels of U38, the viral polymerase, distinguish between plasma viraemia and other forms of HHV6B carriage which are not associated with overt disease. This is turn allows for reverse-transcriptase qPCR assays designed to detect U38 transcript levels. It also suggests a way forward for RNA-seq-based diagnostic studies which detect human herpesviruses within their datasets and hope to distinguish bystander or latent infections from high-level reactivation that is more likely to be associated with disease [78]. Sensitivity and specificity issues currently make introducing metagenomic sequencing for HHV6A/B detection and monitoring into the clinic challenging [79].

#### 2.2.3. Human Herpesvirus 7

Human herpesvirus 7 is perhaps the least studied of the viruses discussed here, and it is not a coincidence given that HHV7 is only weakly linked to specific pathologies, with similar childhood primary infection symptoms as HHV6B (roseola) [80]. HHV7 is lymphotropic, replicates in CD4+ T cells, and is occasionally seen to reactivate in transplant patients where it may be associated with meningitis [81]. It is also found in the CSF of a small proportion (~5%) of adults with neurological disorders [82], and has been tentatively linked to Alzheimer’s disease [83].

The first HHV7 strain to be isolated was RK from a healthy US male [84], but the first to be sequenced was JI [85]. There are only three available genomes to-date, rounded out by the UK sequence UCL-1 [86]. Without a geographically diverse collection of HHV7 genomes, it is difficult to deduce whether currently circulating HHV7 diversity reflects patterns laid down since modern humans migrated Out-of-Africa (as with HSV1 [34]) or more recent genotype replacement since the origins of agriculture [49].

### 2.3. Gammaherpesviruses

#### 2.3.1. Epstein-Barr Virus

Primary Epstein-Barr virus infection usually occurs in childhood and early infection is typically mild or asymptomatic, but in adolescents and adults 25–50% of primary infections show the classic symptoms of sore throat, lymphadenopathy and tiredness [87]. This symptomatic infection is known variously as infectious mononucleosis, glandular fever and ‘kissing disease’ – and transfer of saliva is an important route of infection [88]. EBV is currently the best-represented herpesvirus in Genbank (Table 1), with over 1000 genomes available, including paired blood and tissue/cell-specific genomes. As the first human herpesvirus to have its whole genome sequenced (Figure 1), this seems an appropriate achievement.

EBV’s identity as a tumour virus and its epidemiological association with autoimmune diseases, such as multiple sclerosis, has undoubtedly driven the proliferation in genome sequences (e.g., [89,90,91]), as researchers seek to understand the sequence diversity which associates with particular cancers in different parts of the world [92]. EBV has the advantage that, through the derivation of lymphoblastoid cell lines or target enrichment of salivary DNA, EBV from healthy people can be sequenced and studied almost as easily as the EBV found in tumours and lymphomas [18]. Comparisons of EBV from blood and from specific cell subsets within the same individual have shown that viral as well as host mutations and structural variants play a role in neoplastic proliferations such as chronic active EBV [93].

The field has now sufficiently matured for viral genome-wide association studies (GWAS) to be possible, comparing EBV genomes from cancer cases with those of healthy, location-matched controls [94]; and to study how immune selection pressure has shaped global EBV diversity [20]. Many more EBV genomes are present in cancer genome datasets. For example, recent studies of Burkitt’s lymphoma genomes from endemic and sporadic regions of the world have sequenced the EBV genome as well as the lymphoma genome. This has revealed associations between the presence of EBV and the kinds of mutations present within the tumour [95], and also that EBV type (1 vs 2) influences the tumour’s mutational burden [96]. The assembled EBV genome sequences from these studies are not currently available in GenBank but the raw sequence data represent a resource to be mined in the future.

#### 2.3.2. KSHV

Kaposi’s sarcoma (KS), the skin cancer which gives human herpesvirus 8 its name (Kaposi’s sarcoma-associated herpesvirus) was first described in 1872. The HIV epidemic a century later, with KS as an AIDS-defining illness, suggested a link between KS and an infection [97]. PCR-based techniques then identified the gamma herpesvirus KSHV [98], which is associated with vertical (mother-to-child) transmission in classic and childhood KS, and horizontal (sexual or shared saliva) transmission and AIDS-associated KS in HIV-infected adults [99].

Two years after the virus was discovered, in 1996, the KSHV genome was sequenced using a combination of cosmid and phage library sequencing. This was followed by a trickle of further genomes until the publication of large collections from specific geographic regions provided by Olp and colleagues (Zambia [100]) and Sallah and colleagues (Uganda [101]). Other individual genomes have been sequenced from model cell lines or experimental systems (eg SPEL [102] and JSC-1 [103]), plus a small collection of genomes from Japan [104], but these studies encapsulate the current diversity of KSHV genomes publicly available. A key question is whether patterns of KSHV genetic diversity in living people reflect past demographic processes such as drift and host migration, or whether certain KSHV genotypes have evolutionary advantages that increase their transmissibility or pathogenicity.

### 2.4. Long-Read Sequencing of Herpesvirus Genomes and Getting to Finished Genomes

A number of sequencing platforms provide potentially transformative ways to sequence and study herpesvirus genomes (reviewed in [105]). The two best known are Oxford Nanopore Technologies and PacBio. Long-read genome technologies have been used to assemble alpha [106,107], beta [108] and gamma [109] herpesvirus genomes. Long-read technologies, which allow direct RNA molecule sequencing, are also a popular way to better characterise the transcriptomes of human herpesviruses, such as HSV-1 [110,111]; and VZV [112,113]. The techniques used have recently been reviewed elsewhere [114].

Long-read sequencing platforms have an increased potential to detect structural variants and copy number repeats within herpesvirus genomes, compared to short-read technologies. They are also likely to be important in the future for disentangling the issue of mixed-genotype infection by covering the whole haplotype of interest in a single read [61]. There are, however, still problems for long-read DNA virus sequencing without enrichment. Eckert and colleagues [115] found no on-target HCMV reads when using Nanopore sequencing of HCMV-infected cell cultures without enrichment. Similarly, a study of copy-number repeats in cultured HHV6B strain Z29, used Nanopore sequencing in an attempt to resolve the repetitive regions of the genome, but mapped only two reads partially covering the region of interest [73].

Generating finished or closed herpesvirus genomes using short-read sequencing, particularly when working directly from clinical material, remains challenging. The terminal and internal repetitive regions of herpesviruses are difficult to resolve using short-read sequencing alone [116] as individual reads do not cover the entire region and cannot be accurately mapped to a specific repeat, leading to poor sequence coverage and quality in these regions [54]. There are methods to address this problem: Palser and colleagues resolved the EBV IR1 repeat number for various strains by correlating Southern blot data & normalized read depths [117]. Alternative approaches include trimming [118] or masking repetitive regions as unknown sequence (Ns), or replacing them with the same repeat sequence and/or copy number seen in the reference sequence for that species [119]. It is unclear to what extent current long-read sequencing chemistry will help to resolve this problem. For example, higher error rates (relative to Illumina) and low sequence complexity from Nanopore MinION HSV1 sequence data meant that the long-read data was not enough to resolve the sequence of the internal or terminal repeat regions of clinical strains [107].

The GC content of different genomic regions presents a two-fold problem for herpesvirus sequencing [120]. As the GC content of a region increases, so too does the Illumina error rate, particularly in repetitive regions of eg HSV1 where the GC content may be 90% [120,121]. Target enrichment technologies also perform more poorly as GC content increases above 65–70%, leading to lower coverage of high GC content regions [12], increasing the chances that incorrect bases will be called as the consensus sequence. The correlation between read quality and accuracy, and GC content in Nanopore MinION data is less strict [122]. Genome polishing with long-read technologies may be a successful future direction to avoid problems of GC-related errors, low coverage and poor read mapping.

## 3. Conclusions and Future Directions for Human Herpesvirus Sequencing Studies

The falling cost and increasing use of metagenomic sequencing for infection diagnosis [14] can only reward the field of herpesvirology. Our understanding of the sequence diversity of herpesviruses 6–8 and disease associations of HHV7 may become clearer as increased reporting of herpesvirus genomes identified in clinical metagenomic studies becomes routine [123]. The challenge will then become how to interpret this data, collected without the specific hypotheses many dedicated herpesvirus sequencing projects have, such as the search for drug resistance mutations.

Deep-sequencing and longitudinal sequencing of herpesviruses, particularly in immune compromised patients (e.g., [55,59]) will be important to test hypotheses concerning mixed-genotype infections generated using other methods, such as qPCR genotyping or Sanger sequencing. It is possible that identifying mixed-strain/genotype herpesvirus infections or individuals with high herpesvirus sequence diversity could become a biomarker for more severe disease (e.g., [61,124]).

Where large numbers of herpesvirus genomes are available, as for EBV, HCMV and HSV-1 and -2, virologists increasingly have the ability to compare patterns of diversity and associations of particular genotypes with specific populations at a genomic scale [20]. Genome-to-genome association studies have been used to study the co-evolution of and interactions between HIV [125], *Streptococcus pneumoniae* [126], hepatitis C virus [127] and human genomic variation. By utilising collections of genomes from across the world with detailed metadata, similar analyses are now possible for many of the human herpesviruses.

Human herpesviruses are also finally entering the ancient DNA era [128], with the power to address questions and controversies in their evolution such as the antiquity of currently circulating VZV genotypes [21,49]. For some of the less-sequenced human herpesviruses, such as HHV7, there is a reasonable possibility that more ancient strains of HHV7 may eventually be available, sequenced metagenomically and by chance, than currently circulating strains.

Finally, it is hoped that the geographic representation of particular regions, both in the origin of sequences and the location of research groups with the technology and expertise to conduct herpesvirus sequencing, will continue to expand. Neither patients nor researchers can afford for whole continents to miss out on the insights which genomics can contribute [129].

## Figures and Tables

**Table 1:** The scale of publicly available human herpesvirus genome sequences.

Virus	Whole Genomes in Genbank * (as of 28/08/2019)
Human herpesvirus 1—herpes simplex virus 1	288
Human herpesvirus 2—herpes simplex virus 2	378
Human herpesvirus 3—varicella-zoster virus	247
Human herpesvirus 4—Epstein-Barr virus	1043
Human herpesvirus 5—cytomegalovirus	315
Human herpesvirus 6 (unclassified)	28
Human herpesvirus 6A	91
Human herpesvirus 6B	102
Human herpesvirus 7	3
Human herpesvirus 8—Kaposi’s sarcoma-associated herpesvirus	33

* Not including genomes labelled as *Modified microbial nucleic acid*, *Recombinant viral vector for gene transfer into lymphocyte* or sequences from patents with no metadata. May include independent sequencing of the same isolate or strain by separate laboratories and/or technologies.
